# The Relationship Between Environmental Statistics and Predictive Gaze Behaviour During a Manual Interception Task: Eye Movements as Active Inference

**DOI:** 10.1007/s42113-023-00190-5

**Published:** 2023-11-21

**Authors:** David Harris, Sam Vine, Mark Wilson, Tom Arthur

**Affiliations:** School of Public Health and Sport Sciences, Medical School, University of Exeter, St Luke’s Campus, Exeter, EX1 2LU UK

**Keywords:** Predictive processing, Eye tracking, Gaze, Bayesian, Interception, Computational

## Abstract

Human observers are known to frequently act like Bayes-optimal decision-makers. Growing evidence indicates that the deployment of the visual system may similarly be driven by probabilistic mental models of the environment. We tested whether eye movements during a dynamic interception task were indeed optimised according to Bayesian inference principles. Forty-one participants intercepted oncoming balls in a virtual reality racquetball task across five counterbalanced conditions in which the relative probability of the ball’s onset location was manipulated. Analysis of pre-onset gaze positions indicated that eye position tracked the true distribution of onset location, suggesting that the gaze system spontaneously adhered to environmental statistics. Eye movements did not, however, seek to minimise the distance between the target and foveal vision according to an optimal probabilistic model of the world and instead often reflected a ‘best guess’ about onset location. Trial-to-trial changes in gaze position were, however, found to be better explained by Bayesian learning models (hierarchical Gaussian filter) than associative learning models. Additionally, parameters relating to the precision of beliefs and prediction errors extracted from the participant-wise models were related to both task-evoked pupil dilations and variability in gaze positions, providing further evidence that probabilistic context was reflected in spontaneous gaze dynamics.

This paper considers the optimality principles that underlie dynamic visuomotor control. Strategic shifts of the eyes—via fixations, saccades, and smooth pursuit—are important for acquiring information to guide goal-directed actions (de Brouwer et al., 2021; Zhao & Warren, 2015). This deployment of the visual system is partly driven by mental models of the environment and expectations about the location of salient information (Henderson, 2017; Itti & Koch, 2001). Much of our current understanding of oculomotor control has, however, come from highly constrained tasks requiring simplified motor responses; hence, it is unclear how behavioural outcomes are optimised during more dynamic and naturalistic visuomotor skills, or in the context of more richly structured visual environments (Lappi, 2016). Consequently, some key theoretical questions remain in these contexts. For instance, do eye movements reflect our ‘best guess’ about the likely state of the world, or do they instead minimise errors in a probabilistic way? How does prior knowledge influence gaze control? And what determines when (and by how much) we adapt our visual strategy over successive iterations of an event? The present work aimed to begin answering these questions, by directly evaluating some of the key computational and neuroscientific models of vision that have emerged in recent years (Adams et al., 2012, 2015; Parr et al., 2021).

A body of work has shown that, for many learning and decision-making processes, humans update their beliefs about the world in a statistically optimal way (e.g. Beck et al., 2008; Glaze et al., 2015; Knill & Pouget, 2004; Nassar et al., 2010). Here, ‘statistically optimal’ indicates that all available information is weighted by its reliability—i.e. approximating *Bayesian inference* (Knill & Pouget, 2004). This work has shown that human decision-makers represent uncertain future events much like probability distributions, which describe both the central tendency and the uncertainty (distribution width) of outcomes and dynamically adjust their responses according to both (Körding & Wolpert, 2006). In addition to constantly refining predictions about the world so as to minimise prediction error, Bayesian agents can use actions to further reduce uncertainty in their environment; an idea known as *active inference* (Friston et al., 2016; Parr & Friston, 2019). Rooted in the free energy principle (Friston, 2010; Friston et al., 2006), active inference postulates that humans encode an internal ‘generative’ model of the world, which simulates expected sensory data and infers the likely causes of sensations (Friston et al., 2006; Jiang & Rao, 2022). The same generative model drives motor plans (or policies) that minimise future prediction errors (also known as *expected free energy*; Parr & Friston, 2019). For eye movements, this may entail directing gaze towards parts of the environment that are potentially the *most* surprising or unpredictable, to minimise surprisal in the long run (Itti & Baldi, 2009). Consequently, Friston et al. (2012) conceptualise fixations and saccades as hypotheses about the state of the world (see also Najemnik & Geisler, 2005; Parr et al., 2021). Expected free energy represents not only the minimisation of prediction error (i.e. information gain), but also selection of actions that maximise the probability of outcomes consistent with prior preferences. As such, free energy minimization reflects a trade-off between information gain (epistemic value) and the attainment of preferred outcomes (pragmatic value).

Given the potential explanatory value of active inference as a unified theory of perception and action (Friston, 2010), we sought to test whether eye movements reflect probabilistic predictions about the world during a dynamic visually-guided motor task. There is considerable evidence that eye gaze is, indeed, deployed in a predictive fashion (Hayhoe et al., 2012; Henderson, 2017; Land & McLeod, 2000). For instance, eye movements anticipate the future trajectory of a bouncing ball in a way that is consistent with hierarchical predictive models (Arthur & Harris, 2021; Diaz et al., 2013; Hayhoe et al., 2012; Mann et al., 2019). However, these consistencies do not necessarily mean that visuomotor behaviours are being driven by complex and sophisticated state estimations. Instead, they could reflect a more direct functional coupling between information and movement, where eye movements facilitate prospective control, and are not linked to probabilistic models of the environment (Katsumata & Russell, 2012; Peper et al., 1994). To distinguish between these theoretical positions, it is pertinent to examine the role of contextual uncertainty. Indeed, contrary to these ‘direct perception’ hypotheses, a close correspondence between gaze behaviours and estimated uncertainties would provide evidence in favour of accounts emphasising probabilistic generative models, as they would indicate that an agent is estimating the reliability and/or stability of their surrounding world.

A recent study by Bakst & McGuire (2021) has provided a clear demonstration that dynamic predictive inference is manifested in spontaneous gaze dynamics. In this study, participants were asked to report whether numbers displayed over a range of horizontal locations on a screen were odd or even. Short presentation intervals imposed an implicit need to predict onset location. Stimulus locations were drawn from distributions with shifting central tendencies, as well as differing degrees of uncertainty (widths). Participants showed adaptive learning as predictive eye movements (i.e. location before stimulus onset) were adjusted towards the underlying generative mean. Additionally, pre-stimulus gaze variability was correlated with theoretical levels of uncertainty. These findings are strongly suggestive that eye movements minimise prediction error according to probabilistic generative models, although the highly controlled task conditions could limit generalisability to more naturalistic sensory environments. Therefore, the first aim of this study was to test whether eye movements index probabilistic predictions about the world in a similar way during a more dynamic and naturalistic manual interception task.

Since the stimuli used by Bakst & McGuire (2021) were drawn from moving Gaussian distributions, it is also hard to determine whether eye movements were really minimising prediction error probabilistically, or if instead they were a ‘best guess’ about the next onset location. This question about the sensorimotor system has previously been examined in the context of object lifting. Cashaback et al. (2017) described how predictive grip forces (which are scaled to the expected weight of to-be-lifted objects) could (i) follow a strategy that minimizes prediction error (aka ‘minimal squared error’ (MSE)) or (ii) could be a best guess at the most likely weight (aka ‘maximum a posteriori’ (MAP)). MSE seeks to minimize the squared difference between the observed data and the model’s predictions. So, when lifting an object of unknown mass, this would equate to averaging the grip forces for all possible masses in the known set (weighted by the most probable) and using fingertip forces that would be the ‘least wrong’ on average (but are not necessarily ‘correct’ for any single object). By contrast, MAP, finds the most probable values of the hidden variables, which equates to selecting fingertip forces that are ‘correct’ for the most likely object (but effectively ignores all other possible weights). Cashaback et al. observed that fingertip grip and loading forces minimised prediction error using a MSE strategy, as predicted by Bayesian models of sensorimotor learning (Körding & Wolpert, 2004). We can ask the same question of anticipatory eye movements in a ball-interception task with two distinct stimulus onset locations. Here, an MSE strategy would minimise the error between the placement of the fovea and the likely location of the ball (e.g. positioning gaze part way between two locations in a binary choice task), while a MAP strategy would require looking directly at the most probable ball location. Our second aim was therefore to test whether eye movements during interception followed a MSE or MAP strategy.

To further examine the relationship between predictive generative models and human visuomotor control, it is also prudent to consider neurophysiological responses to surprising observations. The way in which observers encode prediction errors has often been studied using non-luminance mediated changes in pupil diameter, which have been shown to track the probabilistic surprise of new sensory observations (Filipowicz et al., 2020; Hayden et al., 2011; Kloosterman et al., 2015; Lavin et al., 2014). These changes in pupil dilation are linked to central signalling of surprise by the locus coeruleus-norepinephrine system (Joshi et al., 2016; Nassar et al., 2012). Previously, Harris et al. (2022a) reported that task-evoked pupil dilations during an interceptive task tracked the rate at which gaze behaviours were updated trial-to-trial, as estimated via Bayesian learning models. Crucially, this was only the case for learning models fitted to individual responses, not the theoretical levels of surprise from a Bayes-optimal simulation, illustrating the importance of individual differences in the precision weighting of prediction errors. Our third aim was, therefore, to test whether objectively more surprising (unlikely) trials were accurately encoded by individual observers and elicited greater task-evoked pupil responses.

Using a manipulation of probabilistic context during an interceptive task in which a target ball was released from one of two horizontally aligned locations, we sought to test the following hypotheses:

o **H1**—Eye movements will be deployed in accordance with a probabilistic generative model, such that the eye position prior to stimulus onset will track the true generative distribution of the onset location.
o **H2**—Following Bakst & McGuire (2021), we expect increased variability of the pre-onset eye position to be related to probabilistic uncertainty of the projection location.
o **H3**—As outlined in active inference models of oculomotor control (Parr et al., 2021), predictive gaze position will be controlled by a minimal squared error, rather than maximum a posteriori, strategy. If observers use a MSE strategy, predictive gaze position should be more extreme under more strongly biased conditions (e.g. 90% left location compared to 70% left), but if adopting a best guess (i.e. MAP) approach, these conditions should be similar.
o **H4**—We expect participants to accurately encode the probabilistic context of the task, such that greater physiological signalling of surprise (pupillary indices of noradrenergic signalling) will be elicited by events that are theoretically more surprising (in a Bayesian sense).

## Methods

### Design

We used a repeated measures design where all participants took part in five counterbalanced probability conditions: 90/10; 70/30; 50/50; 30/70; and 10/90 left/right distributions.

### Transparency and Openness

We report sample size determination, data exclusions, all manipulations, and all measures in the study. All data and analysis code are available at https://osf.io/tgx6r. Data were analysed using RStudio v1.4.1106 (R Core Team, 2017). The study’s design and analysis plan were pre-registered on the Open Science Framework and can be accessed from https://osf.io/8haen. Any analyses not part of the original pre-registration are specified as exploratory.

### Participants

Forty-one participants (ages 18–44 years, mean = 24.2 ± 7.4; 17 males, 24 females) were recruited from the staff and student population at a UK university. Participants were naïve to the exact aims of the experiment. Three of the 41 participants reported being left-handed. They attended a single session of data collection lasting ~ 45 min and were compensated £20 for taking part. Informed consent was obtained in accordance with British Psychological Society guidelines, and the study received approval from the Departmental Ethics Committee (University of Exeter, UK). The study methods closely adhered to the approved procedures and the Declaration of Helsinki. Data collection was completed between January and May 2022.

The target sample size was based on an a priori power calculation using observed effects from a preceding phase of pilot testing (Harris et al., 2022b). Effects in the range of *ω*^2^ = 0.04–0.08 were observed for the primary dependent variable (predictive eye position) and *ω*^2^ = 0.01–0.02 for secondary variables (task performance and task-evoked pupil dilations). A simulation of observed power across a range of sample sizes was conducted using Markov chain Monte Carlo simulations based on known data variance (simr package for R; Green & MacLeod, 2016). For linear mixed effects models examining the effect of condition, 30 participants were sufficient to detect the smaller effects with more than 85% power. As a conservative estimate, and to account for any potential data loss, we recruited an additional 11 participants. Plots of the power curves, R code, and further details of the calculations can be found in the supplementary files (see https://osf.io/axkjn).

### Task and Materials

We used a visuomotor task that consisted of manual interception of oncoming balls projected from two locations, the probability of which could be systematically controlled (see Harris et al., 2022b). This allowed us to regulate both the likelihood of the projection location *and* when this marginal likelihood changed—i.e. both the expected and unexpected uncertainties of the task (Yu & Dayan, 2005). Participants were given no information about the statistical structure of the task or the optimal strategy, so that any emerging eye movement patterns reflected a spontaneous response to the task structure.

**Fig. 1:**
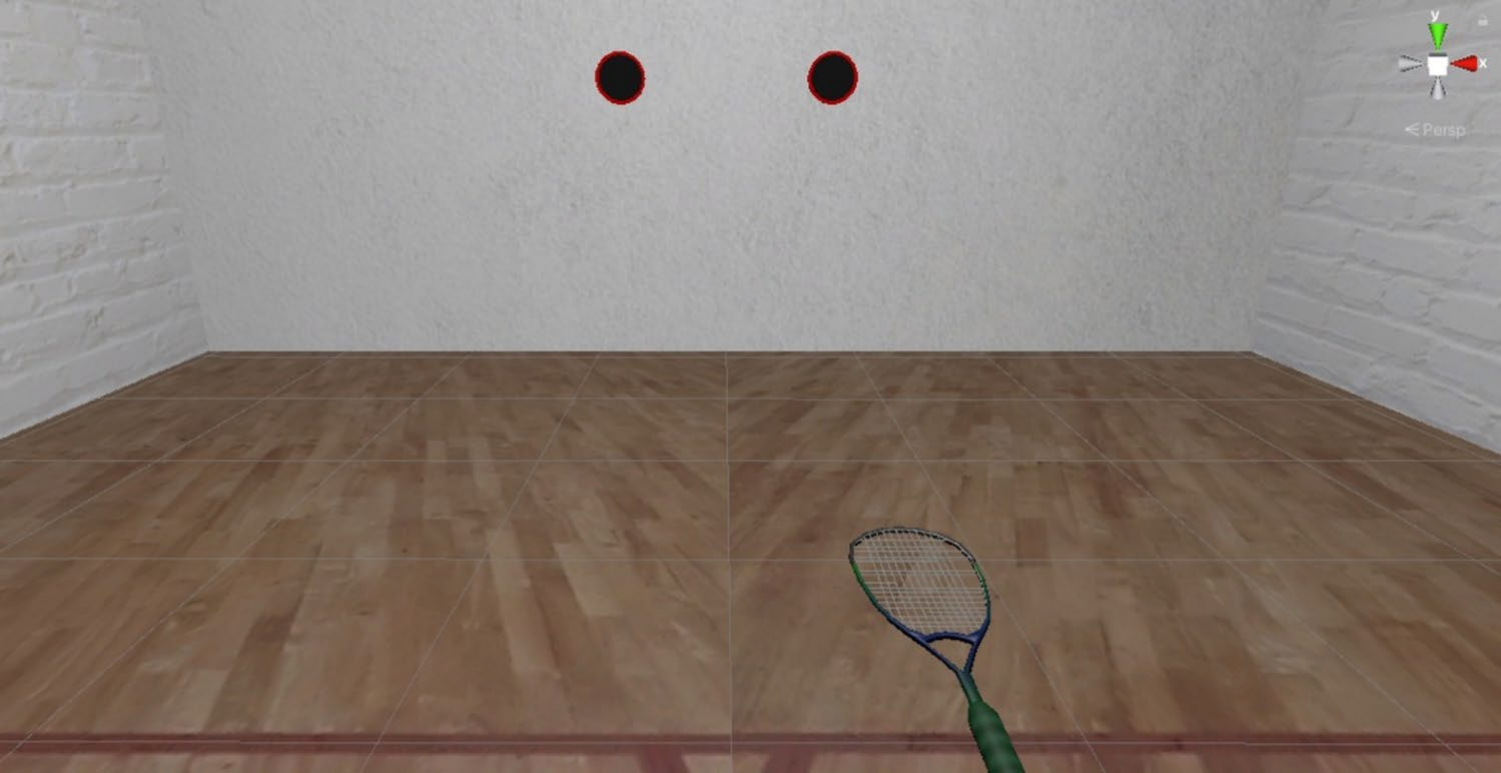
Virtual reality task environment. Note: Participants stood on the red line on the floor. The ball was projected from one of the two locations on the front wall. The ball passed the player without bouncing, and they were instructed to intercept it with the racquet (videos of hit and miss trials of the task are available online: https://osf.io/tgx6r/)

For this task, a virtual environment, simulating an indoor racquetball court, was developed using the gaming engine Unity (v2019.3.1f; Unity Technologies, San Francisco, CA) (see Fig. 1). The VR environment was presented on an HTC Vive head-mounted display (HTC Inc., Taoyuan City, Taiwan) a high-precision, consumer-grade VR system which has proven valid for small-area movement research tasks (field of view 110°, accuracy 1.5 cm, jitter 0.5 mm, latency 22 ms; (Niehorster et al., 2017)). Two ‘lighthouse’ base stations projecting infrared light act as a reference point to record movements of the headset and hand controller at 90 Hz. The headset features inbuilt eye-tracking, which uses binocular dark pupil tracking to monitor gaze at 120 Hz (spatial accuracy 0.5–1.1°; latency 10 ms, headset display resolution 1440 × 1600 pixels per eye). Gaze was calibrated over five virtual locations prior to each condition and upon any obvious displacement of the headset during trials.

The task consisted of a simplified racquetball game where participants were instructed to intercept a ball projected from one of two possible locations at the front of the court using a virtual racquet operated by the Vive hand controller. Balls were 5.7 cm in diameter and resembled the visual appearance of a real-world tennis ball. The visible racquet in VR was 0.6 × 0.3 × 0.01 m, although its physical thickness was exaggerated by 20 cm to facilitate the detection of ball-to-racquet collisions.

### Procedure

Participants attended the lab for a single visit lasting ~ 45 min. They first completed an informed consent form and were fitted with the VR headset. The in-built eye trackers were calibrated at the start of the experiment and on any obvious displacement of the headset. Participants first completed six practice trials (50/50 left/right split) to familiarise themselves with the task. On each trial, participants begun in the centre of the court. The appearance of each ball was cued by three auditory tones which took 2 s to play. The tones were followed by a variable onset delay, whereby the ball was projected during a 0–5-s window. The onset delay on each trial was randomly selected from a uniform distribution. The inclusion of the variable ball onset was based on previous pilot testing, as it created an additional source of uncertainty and a greater implicit demand on correctly predicting the ball origin. The ball was projected to either the left or right side of the participant and reached them on the full (i.e. without bouncing) at around chest height (1.36 m). It took 350–400 ms for the ball to reach the participant, leaving little time to make gaze shifts after released, again placing a demand on correct prediction. When participants intercepted the ball with the racquet, the ball disappeared and a pleasant ‘ding’ sound was played, alongside a haptic vibration from the handheld controller. If the ball was missed, a ‘buzz’ sound was played.

Participants completed a further six blocks of 20 trials each, which were split across the different probability conditions (10/90; 30/70; 50/50; 70/30; 90/10) and balanced across left and right biases. Trial orders were pseudo-randomised within each block and presented in one of two counterbalanced orders. Twenty trials per block were chosen as it has been shown that subjects learn the value of the mean of a prior distribution within 10 trials (Berniker et al., 2010).

### Measures

#### Predictive Gaze Behaviours

The gaze-in-world coordinates (i.e. intersection point of the gaze vector with the environment) recorded from the VR eye trackers were denoised with a three frame moving median filter and then a second-order 15-Hz lowpass Butterworth filter (Fooken & Spering, 2020). From this, we calculated the following measures of predictive gaze behaviour:

i) *Predictive gaze location* was defined as the horizontal gaze position at the termination of the auditory tones (averaged over a 50-ms window). As the ball could appear at any time after the tone, this was taken as the most critical moment for anticipating the projection location and the variable that most closely matched the predictive gaze variable from Bakst & McGuire (2021).
ii) *Mean pre-release position* was defined as the average of the horizontal gaze position coordinates during the time window from the first beep until the ball release. This therefore reflects the visual search of the projection space for the whole pre-stimulus period (e.g. Figure 3D) and therefore the locations that were deemed most salient.
iii) *Gaze variability* was calculated to assess whether variance in gaze position was related to precision of predictions. Following Bakst & McGuire (2021), this was defined as the cumulative distance travelled by the gaze-in-world coordinates before stimulus onset (i.e. the 2-s window from the start to the end of the tones), using the Euclidean distance between successive points.

#### Pupil Dilation

Task-evoked pupil dilation indexes the neurophysiological response to probabilistically surprising events (Harris et al., 2022a; Joshi & Gold, 2020; Nassar et al., 2012). We therefore used trial-wise changes in pupil diameter to compare whether statistically unlikely trials were experienced as surprising by participants, i.e. were they accurately encoding the probabilities. Binocular pupil diameter (in millimetres) was recorded at 90 Hz from the in-built eye tracking system in the VR headset. The data were processed using protocols well established in the literature (Relaño-Iborra & Bækgaard, 2020). Firstly, blinks were identified from portions of the data where the pupil diameter was 0, before being removed, padded by 150 ms, and replaced by linear least-squares interpolation (Lemercier et al., 2014; Relaño-Iborra & Bækgaard, 2020). The resulting signal was then filtered using a low-pass Butterworth filter with 10-Hz cut-off. We performed a baseline correction to account for fluctuations in arousal (as recommended by Mathôt & Vilotijević, 2022) by subtracting the pupil size during a 2000-ms window before stimulus onset from the peak pupil response over the trial (from ball release until 1000 ms after the ball reached the player).

### Computational Modelling

In addition to the pre-registered analyses, we used computational modelling to examine whether eye movements followed an active inference (i.e. Bayesian) strategy. We tested whether trial-to-trial changes in eye position were better explained by simple reinforcement learning or Bayesian inference principles. To do this, we compared how accurately two associative learning models—the Rescorla-Wagner (R-W) learning rate model (Rescorla & Wagner, 1972) and the Sutton K1 model (Sutton, 1992) —fit our data, compared to Mathys and colleagues’ hierarchical Gaussian filter (HGF) model of Bayesian inference (Mathys et al., 2011, 2014). More detailed descriptions of these approaches to human learning are provided in the supplementary files (https://osf.io/yadu6), but in short, both associative learning models assume that beliefs about a value (*v*) are updated over trials (*k*) in proportion to the size of the preceding prediction error (*δ*) and a stable learning rate scalar (*α*), which in the Sutton K1 is further weighted by recent prediction errors. The HGF instead characterises the learning of a parameter value as achieved through hierarchical representations of probabilities that encode beliefs about the world, the (un)certainty of those beliefs, and how likely the world is to change (Mathys et al., 2014; Yu & Dayan, 2005). As a result, rates of belief updating are adjusted dynamically according to uncertainty about observations and the wider unpredictability of the environment, which is not the case for the R-W or Sutton K1 models.

In the HGF, changing beliefs about a state of the world (*x*) are modelled as a ‘Gaussian random walk’, which describes the evolution of a time series via a Gaussian probability distribution over *x*. In the current context, *x* refers to a belief about the likely release location of the target ball. While this outcome (left/right) is binary, we model an agent’s belief about it using a Gaussian distribution (described by the mean and variance), which evolves from the posterior estimate at the preceding time point. The values of *x* can be described as follows:

$${x}^{(k)}\sim N\left({x}^{(k-1)},\vartheta \right), k=\mathrm{1,2},\dots$$

where *k* is a time index, *x*^(k−1)^ is the mean of the distribution, and ϑ is the variance at the preceding time point. The value of *x* at time *k* will then be normally distributed around its values at *k*-1. This is effectively a two-level HGF model, consisting of the observed instances of *x* and the agents evolving beliefs about *x*. However, the HGF approach also allows us to model how learning about the parameter *x* might be faster or slower depending on how changeable the environment is perceived to be. Therefore, instead of using a fixed variance parameter (ϑ), we can employ a variance parameter that may itself also vary (or ‘walks’), creating a three-level HGF (see Fig. 7 for a schematic representation). This effectively models a state where the observer is not only representing how a state changes over time, but how its rate of change might itself change (i.e. its volatility). By replacing the fixed ϑ parameter with a function of a second hidden variable *x*_2_, so that *x* becomes *x*_1_ and we create a hierarchical model:

$${x}_{1}^{(k)}\sim N\left({x}_{1}^{(k-1)},f({x}_{2})\right), k=\mathrm{1,2},\dots$$

This hierarchical model can be further extended so that the rate of change of the volatility can itself change over time (creating a four-level model), and so on. These additional hierarchical levels enable us to model more complex human learning in shifting environmental contexts (see Arthur et al., 2023 for an example with sensorimotor tasks).

An agent’s responses (looking to the left or right) are taken to be a function of their evolving belief about the release location (*x*). The agent’s action (eye position) is assumed to be a Gaussian distribution around the inferred mean of the relevant state (*x*). The parameter ζ quantifies the noise of this distribution, which effectively controls the extent to which mapping from beliefs to responses is fully deterministic or more exploratory.

Our modelling approach followed the ‘observing the observer’ framework (Daunizeau et al., 2010), in which Bayesian inference is used to estimate the inference processes of the agent (participant). Each learning model consists of two components, a perceptual model and a decision or response model. The perceptual model is used to estimate the agent’s perception of their environment (posterior estimates), while the response model estimates the mapping between beliefs and observed actions. When both observations (*u*) and responses (*y*) are known, the intervening learning parameters can be estimated. Observations (*u*) in the models were the onset location of the ball on each trial, and responses (*y*) were the eye position (i.e. predictive gaze location). All models contained free parameters that could vary to accommodate the observed data that we wished to model. These parameters were optimised using maximum-a-posteriori estimation to provide the highest likelihood of the data given the model and parameter values. For associative learning models, the free values were beliefs about onset location and learning rate, which were set at a neutral starting value and given wide variance. For un-bounded parameters in the HGF models, we chose prior means that represented values under which an ideal Bayesian agent would experience the least surprise about its sensory inputs. As such, they were based on a running a simulation with the real sequences from the experiment. The priors were given a wide variance to make them relatively uninformative and allow for substantial individual differences in learning (for additional details on starting priors, and tests of parameter recoverability and identifiability, see Table 1 and https://osf.io/tgx6r/).

**Table 1:** Prior means and variances of the perceptual models

	Prior mean*	Prior variance
Two-level HGF		
κ**	1	0
ω	− 5.9	8
ϑ	− 4	0
μ_2_	0	8
σ_2_	0.1	1
μ_3_	1	0***
σ_3_	1	1
Three-level HGF		
κ**	1	0
ω	− 5.9	8
ϑ	− 4	8
μ_2_	0	8
σ_2_	0.1	1
μ_3_	1	8
σ_3_	1	1
Four-level HGF		
κ**	1	0
ω	− 5.9	8
ϑ	− 4	8
ϑ_2_	− 2	8
μ_2_	0	8
σ_2_	0.1	1
μ_3_	1	8
σ_3_	1	1
μ_3_	1	8
σ_3_	1	1
R-W model		
α	0.5	1
*v*	0.5	1
Sutton K1 model		
*h*	0.5	1
*v*	0.005	16

*The HGF class prior means were determined by running a Bayes-optimal simulation of the task (where the variances were set wide to account for individual differences) and taking the resultant posterior means as starting values here (Mathys et al., 2011). **Kappa, which allows a variable strength of coupling between levels, was fixed to reduce model complexity in light of the relatively few trials. ***Constraining the variance of the third level prevents any influence of volatility, making this act as a two-level model

The HGF toolbox (Mathys et al., 2011, 2014) from the open source software package TAPAS (which can be downloaded from http://www.translationalneuromodeling.org/tapas; (Frässle et al., 2021)) was used for model fitting and comparison routines. Bayesian model selection (Rigoux et al., 2014) was then used to compare model fits, using the spm_BMS.m routine from the SPM12 toolbox (https://www.fil.ion.ucl.ac.uk/spm/ software/spm12/). Bayesian model selection estimates the probability that a given model outperforms all others in the comparison (the ‘protected exceedance probability’), effectively treating the model as a random variable that could differ between participants.

### Data analysis

Data processing was performed in MATLAB 2022b (MathWorks, USA) using bespoke analysis scripts, all of which are available from the Open Science Framework project page (https://osf.io/gprbu/). For the between-condition comparisons (i.e. the non-model-based analyses), the first ten trials from each condition were excluded as there was no basis for a reliable prediction (given that participants require around ten trials to learn the true distribution; Berniker et al., 2010). Statistical analysis was performed in Rstudio v1.4.1106 (R Core Team, 2017). A series of linear mixed effects models (LMMs; fitted using restricted maximum likelihood in the lme4 package (Bates et al., 2014)) were used to examine the pre-registered hypotheses. Model fit checks were performed using the ‘performance’ package (Lüdecke et al., 2021) and can be accessed from the supplementary materials (https://osf.io/p95w6). Our analysis approach sought to allow for different baseline values for each participant as well as different effects of ‘condition’ for each participant. For each dependent variable, we therefore compared a model with only random intercepts for the ‘participant’ factor (i.e. DV ~ IV + (1|Participant)) with a model that included both random intercepts and random slopes (i.e. DV ~ IV + (1 + IV|Participant)) to determine the best fitting model. The model with the lower Akaike information criterion (AIC) value was chosen in each case. We report *R*^2^ and standardised beta effect sizes for the mixed effects models and follow Acock’s (2014) rule of thumb for std. beta that < 0.2 is weak, 0.2–0.5 is moderate, and > 0.5 is strong.

## Results^1^

### Does the Position of Gaze Prior to Stimulus Onset Track the Central Tendency of the Underlying Generative Distribution (H1) and Does It Indicate a MSE or MAP Strategy (H3)?

**Fig. 2:**
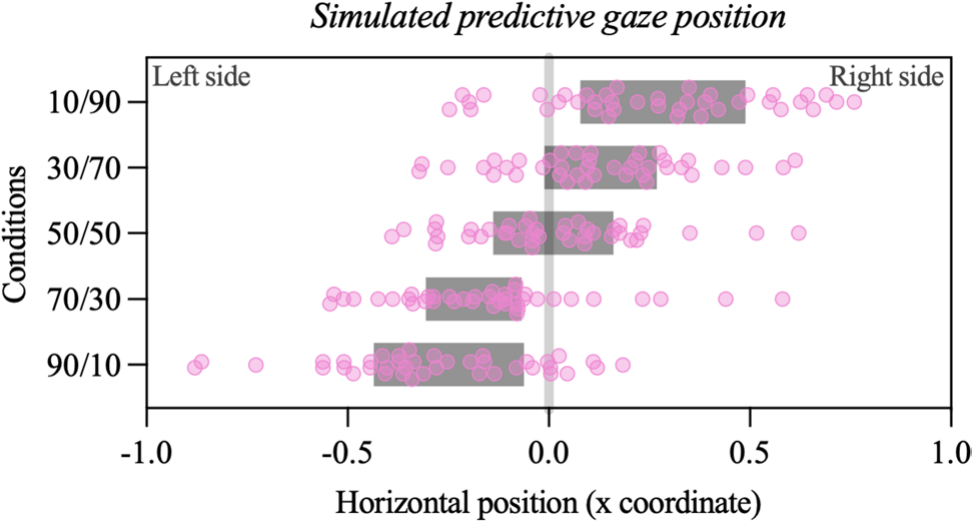
Model-based simulations of predictive gaze behaviour in the experimental task. Note: To help motivate our hypotheses and determine exactly the behaviour that a Bayes-optimal observer would display in this task, we ran a series of simulations using a model of Bayesian inference (the hierarchical Gaussian filter (Mathys et al., 2011, 2014), described in detail in the ‘Methods’ section). We provided 40 simulated observers with the exact trial order used in the study and recorded their trial-by-trial responses. We allowed a degree of randomness/noise in the responses of the simulated agents to provide some variance in the data, but even so these simulated responses are likely to be less variable than real eye movements. The simulations provided a clear demonstration that for Bayes-optimal observers, mean gaze position would be more extreme under increasingly biased conditions. Grey boxplots indicate the interquartile range, with overlaid data points. Full details of the modelling approach and the MATLAB code are provided in the supplementary files (https://osf.io/cjkz7)

**Fig. 3:**
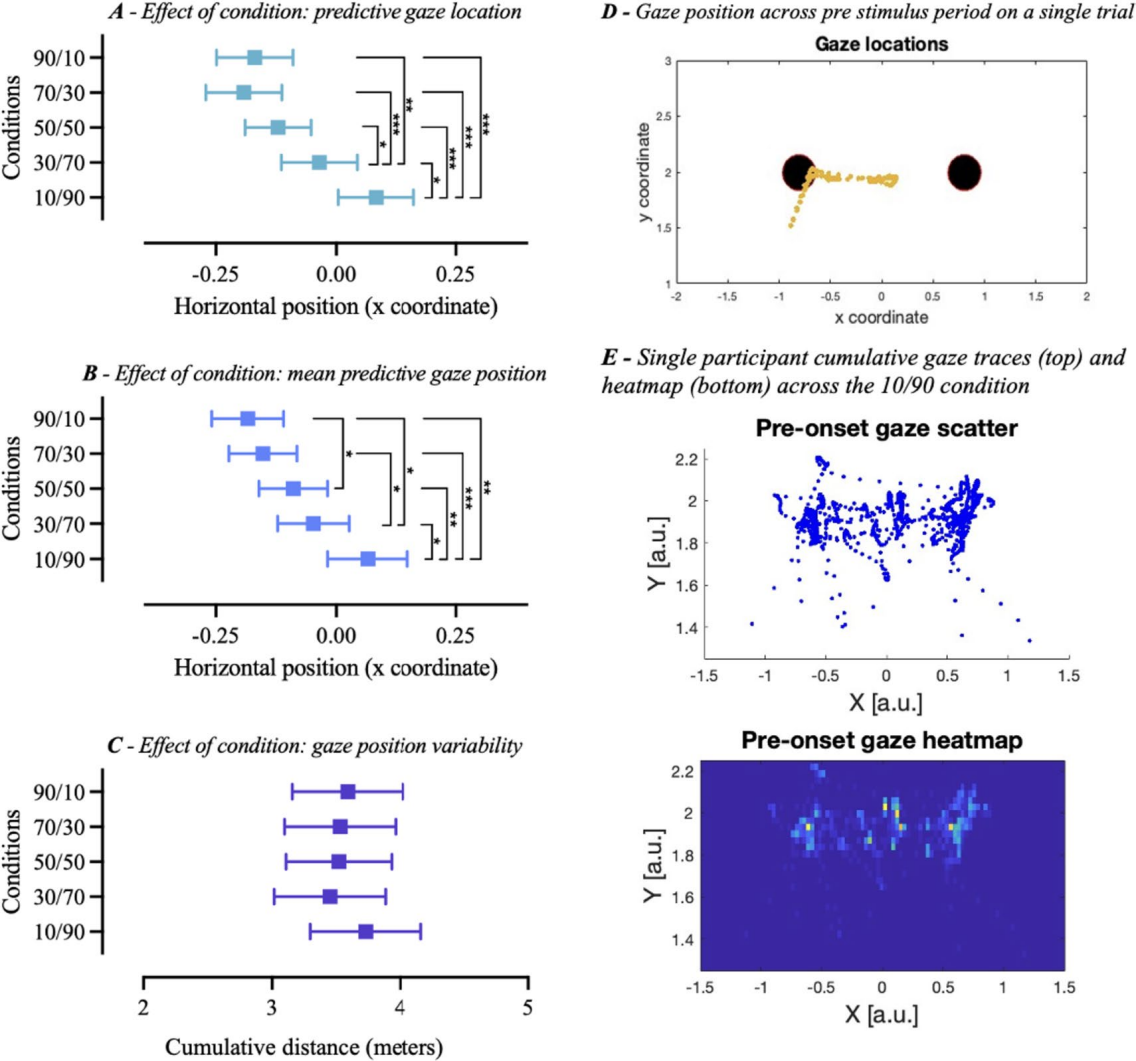
Predictive gaze behaviour results. Note: **A** Predictive gaze location across conditions (estimated marginal means and 95% CI error bars). Significant effects are indicated by an asterisk. **B** Mean predictive gaze position across conditions (estimated marginal means and 95% CI error bars). **C** Plot of gaze variability across conditions (estimated marginal means and 95%CI error bars), calculated as the cumulative distance travelled in coordinate units (equivalent to meters). **D** and **E** show examples of gaze position for a single trial (**D**) and over a whole condition (**E**). *p < .05, **p < .01, **p < .001, a.u. = arbitrary units

To examine if eye movements were indeed deployed in accordance with a probabilistic internal model (H1) and tracked the generative distributions in the environment (corresponding to the model-based predictions in Fig. 2), we compared the predictive gaze location and the mean pre-release position (from the start of the auditory tones to ball release) across conditions. The mixed effects model (see Fig. 3A) for predictive gaze location (with random intercepts for participant) had a conditional *R*^2^ of 0.12 and marginal *R*^2^ of 0.03. All condition effects were significant relative to the reference condition (10/90) (*p*s < 0.002, std. betas > 0.20) and tracked the pattern of simulated responses (see Fig. 1). Pairwise comparisons with Bonferroni-Holm adjustment indicated significant differences between all conditions (*p*s < 0.04) except for 90/10 leftward bias compared to 70/30 (*p* = 0.56) or 50/50 (*p* = 0.30), or between 70/30 and 50/50 (*p* = 0.10).

The model for mean pre-release gaze position (with random slopes and intercepts for participant) had a conditional *R*^2^ of 0.50 and marginal *R*^2^ of 0.06 (see Fig. 3B). All condition effects were significant relative to the reference condition (10/90) (*p*s < 0.001, std. betas > 0.36). Pairwise comparisons with Bonferroni-Holm adjustment indicated statistically significant differences between all conditions (*p*s < 0.03) except for 30/70 v 50/50 (*p* = 0.10), 50/50 v 70/30 (*p* = 0.08), and 70/30 v 90/10 (*p* = 0.35).

### Does the Variability of the Pre-onset Eye Position Track the Probabilistic Uncertainty of the Projection Location (H2)?

**Fig. 4:**
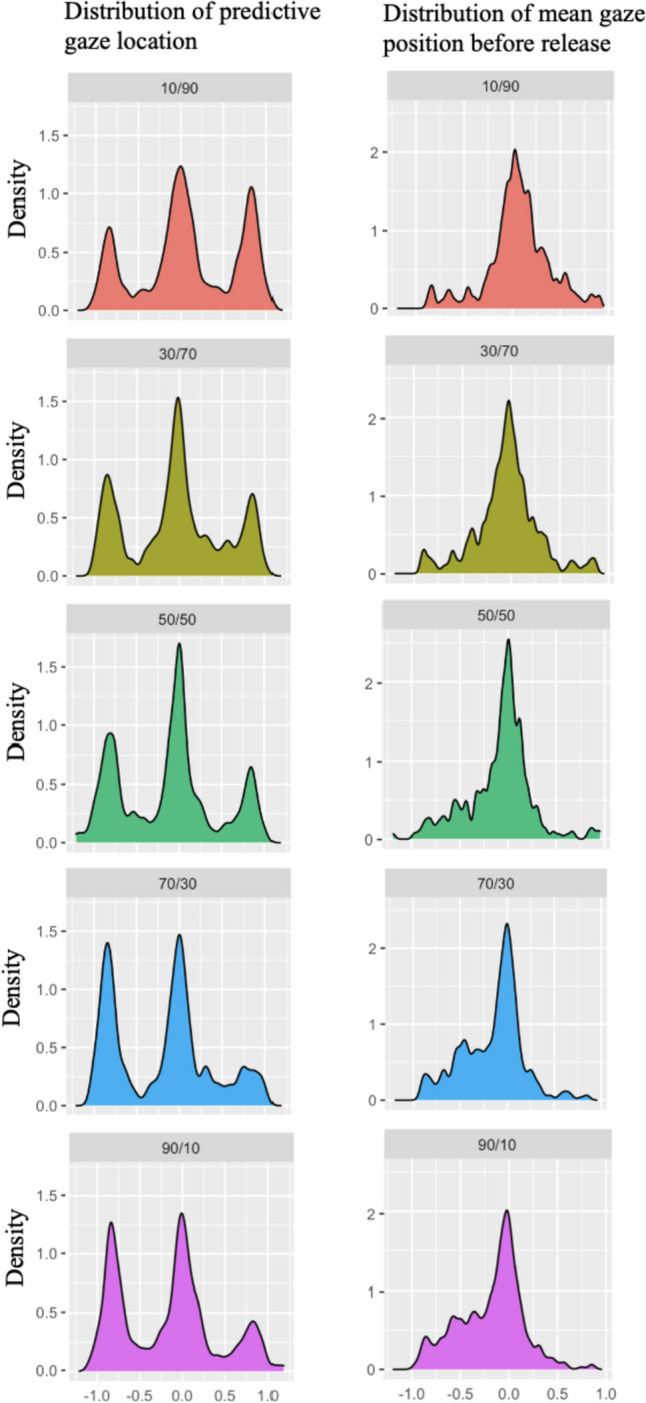
Distributions of predictive gaze location (left) and mean gaze position before release (right)

To test whether variability in pre-onset eye position was related to probabilistic uncertainty, as reported by Bakst & McGuire (2021), we fitted a linear mixed model to the gaze variability measure (cumulative distance travelled). The model (with participant as a random effect) had a large conditional *R*^2^ of 0.30 but very small marginal *R*^2^ of 0.002. Compared to the reference category (10/90 condition), only the 30/70 condition was significant (*p* = 0.03, std. beta = 0.13) (see Fig. 3C). All other condition effects were non-significant (*p*s > 0.06; std. betas < 0.09). No pairwise comparisons were statistically significant following Bonferroni-Holm adjustment (*p*s > 0.34), which indicates that gaze variability did not track the precision of predictions (Fig. 4).

### Does Physiological Signalling of Surprise Accurately Encode the Probabilistic Context of the Task (H4)?

**Fig. 5:**
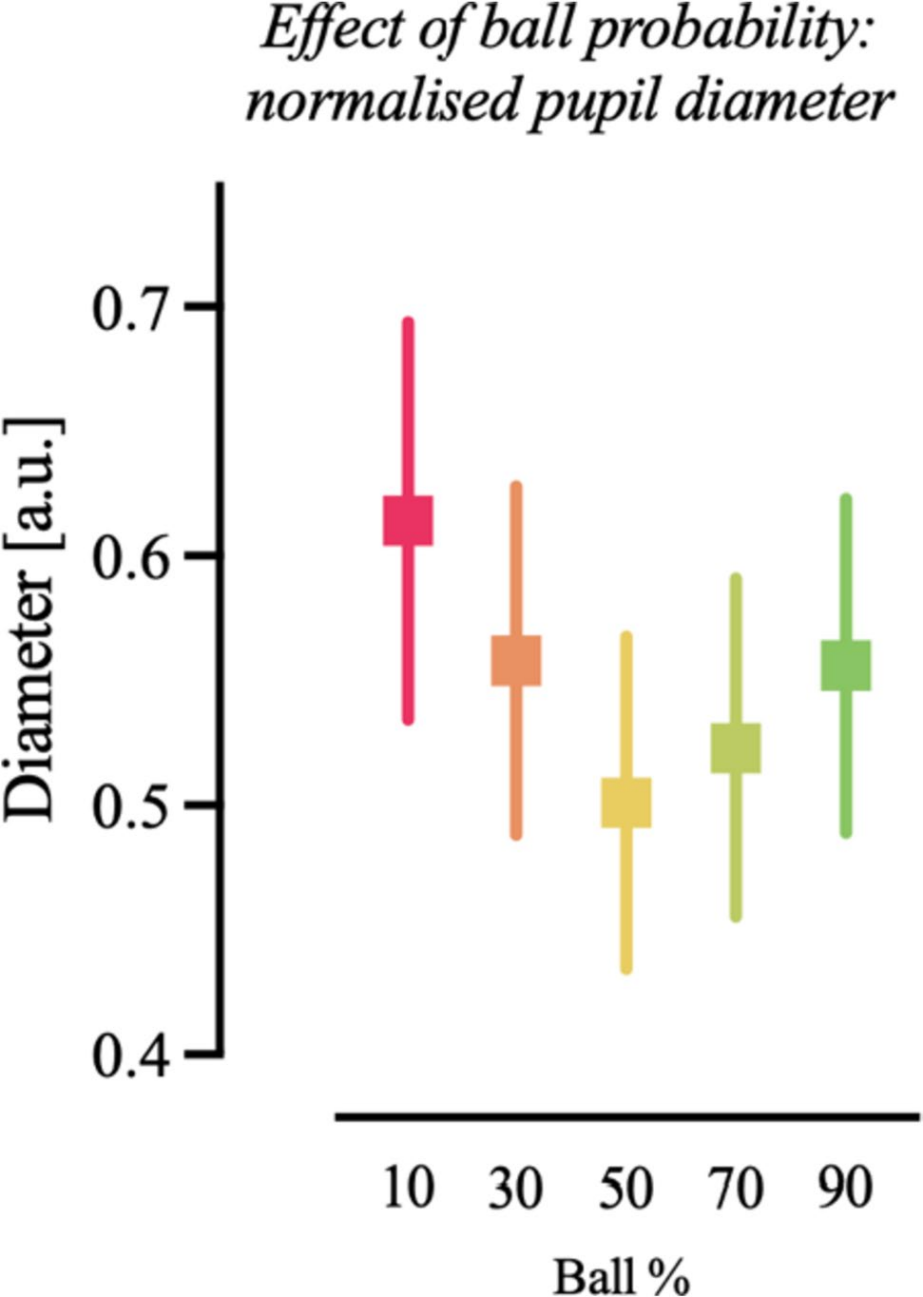
Pupillometry results. Note: Factor level means and 95% CIs

To test whether participants exhibited larger pupillary surprise responses to less probable ball onset locations, as would be the case if they were accurately encoding the probabilistic context of the task (H4), we fitted a linear mixed model (with participant as a random effect) to the task-evoked pupil response (normalised peak dilation). We entered the ball probability as the predictor (i.e. as a factor with levels of 10%, 30%, 50%, 70%, 90%, depending on the probability spilt of the block). The model had a large conditional *R*^2^ of 0.54 and marginal *R*^2^ of 0.01. Relative to the reference category (50%), all levels were significant (*p*s < 0.02, std. betas > 0.10) (see Fig. 5). Pairwise comparisons with Bonferroni-Holm adjustment indicated that, as predicted, pupil dilations were significantly larger for 10% than 50% (*p* < 0.001), 30% than 50% (*p* < 0.001), and 10% than 70% (*p* = 0.004). Unexpectedly, dilations for 90% balls were also significantly larger than for 50% (*p* < 0.001). No other pairs remained significant after the multiple-comparison correction (*p*s > 0.10).

### Computational Modelling

We compared five potential learning models to determine whether trial-to-trial adjustments in gaze position were best explained by Bayesian inference or simple associative learning. Three versions of the HGF with different numbers of levels (two levels (HGF2), three levels (HGF3), and four levels (HGF4)) were compared to the two associative learning models (Rescorla-Wagner (R-W) and Sutton K1 (SK1)). The functional difference between the three versions of the HGF models is whether the random walk of the parameter *x* (i.e. release location) had a fixed variance (HGF2), had an additional level encoding changes of the variance parameter (i.e. a volatility level—HGF3), or even had a further level representing changes in volatility (HGF4). In behavioural terms, this relates to whether uncertainty beliefs remained stable over the course of each block, or whether they were moderated by further high-level beliefs (about whether the environment itself is changing and whether these changes are regular or unstable over time).

**Fig. 6:**
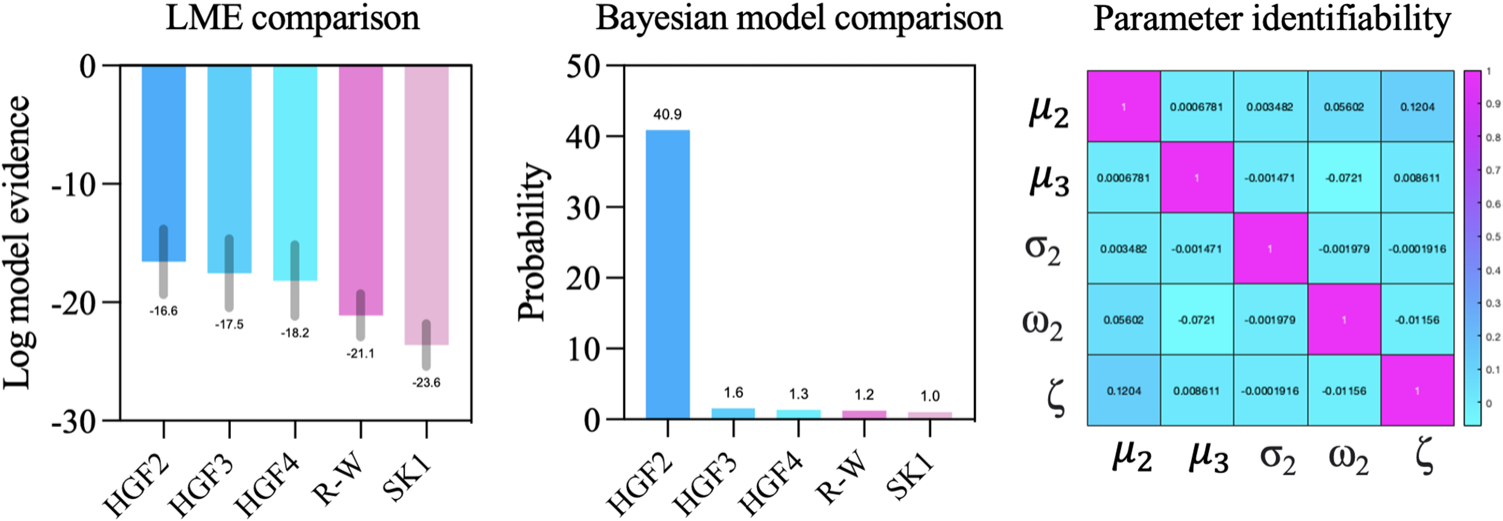
Model comparison and parameter identifiability results. Note: Left: Plot of the log-model evidence (LME) for all models. Middle: Plot of probabilities of the different models in the participant population based on Bayesian model selection, where HGF2 was the most likely generative structure. Right: Parameter identifiability matrix (correlation matrix) for the HGF2, which indicates that no model parameters were highly correlated (i.e. one could not simply be substituted for another)

Results of the model fitting and comparison showed that the HGF2 (see Fig. 8 left for schematic of HGF with two and three levels) was the most likely model, with the highest log-model evidence (see Fig. 6, left), the highest probability (40.9, see Fig. 6, middle) and a protected exceedance probability of 1.00. Bayes factors calculated from the exponential of the differences in the log-model evidence showed the HGF2 was 2.6 times more likely than the HGF3, 4.9 times more likely than the HGF4, 92.7 times more likely than the R-W and 1128.8 times more likely than the SK1.

The winning two-level HGF is effectively a Kalman filter (Kalman, 1960), an algorithm for optimal statistical inference under uncertainty, but which assumes environmental stability. Additional hierarchical levels of the HGF enable shifts in environmental uncertainty (i.e. volatility) to be more effectively modelled, which are not easily accounted for by the Kalman filter (although see Piray & Daw, 2020, for an extension to volatile environments). The better fit of the HGF2 indicates that learning in this task was best described by Bayesian inference rather than associative learning, but that it did not require additional hierarchical levels to account for volatility.

Finally, we explored whether task-evoked pupil responses and gaze variability tracked parameters from the fitted learning models. If pupil dilation and gaze position are both indicators of the encoding of uncertainty, as has been suggested previously (Bakst & McGuire, 2021; Harris et al., 2022a), then both should correlate with uncertainty-related parameters from the participant-wise fitted models (but not beliefs themselves). The simple condition comparisons indicated that pupil responses partially followed the uncertainty of the conditions, but that gaze variability did not. Examining their relationship with personalised learning models could provide more sensitivity to individual differences in learning (Harris et al., 2022a). Following previous studies that have examined the relationship between HGF model parameters and psychophysiological variables (Filipowicz et al., 2020; Lawson et al., 2021), a series of robust linear regression analyses (due to the heavy-tailed distributions of the HGF parameters) were run to obtain individual β weights for the relationship between model parameters and pupil dilation on an individual basis. We then examined whether β weights significantly differed from zero using one-sample *t*-tests for each of the variables of interest.

Pupil dilations were found to have no relationship with the evolving belief trajectory about release location [mu_2_; *t*(40) = 0.11, *p* = 0.92, *d* = 0.02], but were related to both the precision of beliefs [sa_2_; *t*(40) = 5.01, *p* < 0.001, *d* = 0.78] and the rate of change of beliefs [om_2_; *t*(40) = 5.83, *p* < 0.001, *d* = 0.91] (see Fig 8). In line with these results, there was also a relationship with model-estimated precision weighted prediction errors, although this test did not reach significance [*t*(40) = 1.97, *p* = 0.055, *d* = 0.31]. Pre-onset gaze variability on each trial was again unrelated to beliefs themselves [mu_2_; *t*(40) = 0.00, *p* = 1.00, *d* = 0.00]. However, as reported by Bakst & McGuire (2021), the metric was related to the variance (inverse precision) of beliefs [sa_2_; *t*(40) = 2.96, *p* = 0.005, *d* = 0.46], as well as the rate of change of beliefs [om_2_; *t*(40) = 3.76, *p* < 0.001, *d* = 0.59]. No relationship with precision-weighted prediction errors was observed [*t*(40) =  − 0.21, *p* = 0.83, *d* =  − 0.03].

**Fig. 7:**
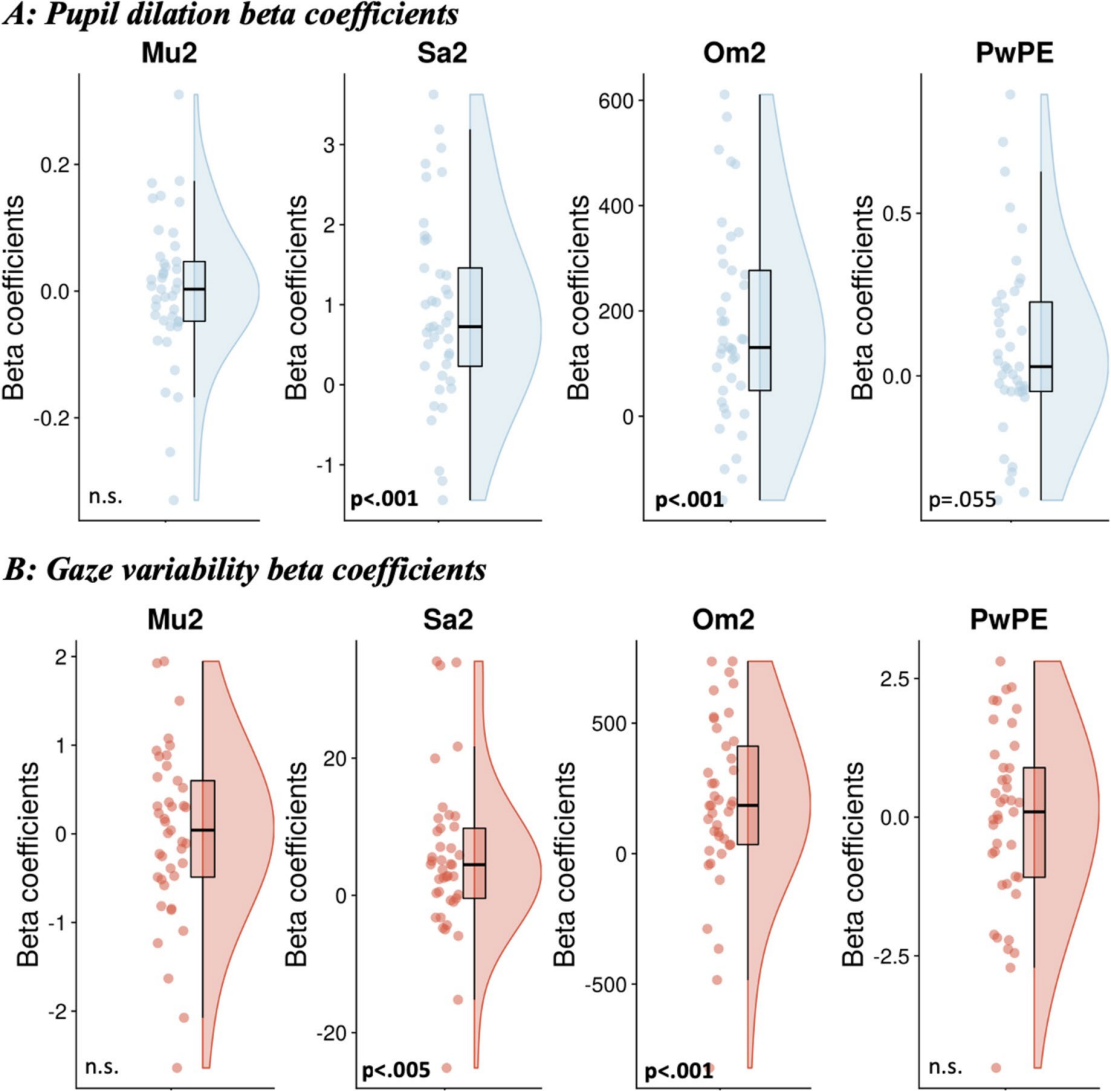
Beta coefficients for relationships of model parameters with gaze variability and pupil dilations. Note: Raincloud plots of the beta coefficients from the participant-wise robust regressions for beliefs (Mu2), precision of beliefs (Sa2), variance of the random walk in beliefs (Om2), and precision-weighted prediction errors (PwPE) shown for pupil dilation (**A**, top) and gaze variability (**B**, bottom)

## Discussion

In this paper, we examined how performers attempt to optimise oculomotor control in a dynamic interception task and whether eye movements minimise prediction error in a fully optimal probabilistic manner (minimal squared error strategy) or, instead, reflect a ‘best-guess’ about the likely state of the world (maximum a posteriori strategy). We tested recent neurocomputational accounts that appeal to the idea of Bayesian inference to explain the deployment of the visual system (Friston et al., 2012; Parr et al., 2021). In summary, our results indicated that participants did encode the probabilistic relationships of the task as would be the case if eye movements were controlled by a generative model (Parr et al., 2021). However, eye movements did not fully correspond with Bayesian inference principles and instead suggested possible trade-offs between uncertainty reduction and predicted action outcomes, or potentially the use of more explicit gaze strategies. This work represents an important development in the empirical testing of active inference theories in the context of more complex movement behaviours, which contrasts with a focus on binary behavioural choices (Smith et al., 2020) or simple motor or oculomotor movements (Adams et al., 2015; Limanowski & Friston, 2020) in previous work.

Or first hypothesis (H1) was that eye movements prior to stimulus onset would be deployed in accordance with a probabilistic generative model and would therefore reflect the true generative distribution of the onset location. This hypothesis was supported. For both the predictive gaze location and the mean pre-release position, participants controlled their gaze in accordance with the underlying probabilities of the experimental conditions. While not all pairwise comparisons were statistically significant, the overall pattern was plainly similar to the Bayesian learning model predictions in Fig. 2, suggesting that oculomotor control was, at least in part, governed by a generative model that (i) encoded the probabilities of the task environment and (ii) directed the visual system accordingly. This finding is in line with previous studies showing that eye movements index predictions about the world, which are derived from prior experience (Arthur & Harris, 2021; Bakst & McGuire, 2021; Diaz et al., 2013; Friston et al., 2012; Harris et al., 2022a; Vater & Mann, 2021).

As is evident from the plots in Fig. 3A and B, there was a clear leftward bias in gaze position, despite the overall pattern approximating the Bayes-optimal simulations (Fig. 2). This asymmetry likely reflects the greater interceptive challenge on the left side (i.e. a ‘backhand’ shot was required on this side for most participants), which participants tried to counter by biasing their visual attention towards it, presumably making the action easier to execute.^2^ This bias in gaze position suggests a possible trade-off between making statistically accurate predictions about onset location and making predictions that maximise the chance of success. While this result was not anticipated, this trade-off is explicitly predicted by active inference and is referred to within a free energy framework as an ‘epistemic’ versus ‘pragmatic’ trade-off (Friston, 2010; Parr & Friston, 2019). In short, under the free energy principle actions serve to (i) fulfil current goals (aka prior preferences) and (ii) reduce uncertainty. When uncertainty is high, information gathering is prioritised, but as uncertainty is reduced, the achievement of prior preferences is prioritised. The biasing of eye movements to the left reflects a balancing of the epistemic value of predicting correctly, versus the pragmatic value of successfully intercepting the ball (based on predictions about action outcomes). Ecological and affordance-based theories of perception and action also emphasise this tight interplay of vision with movement capabilities, asserting that a primary function of vision is to allow actors to see the world in terms of what they can and cannot do (Fajen, 2007; Katsumata & Russell, 2012). From a wider theoretical perspective, this result highlights the complexity of studying perception and action in more realistic and dynamic tasks, where action capabilities will influence action policy selection in a way that is fundamentally different to simple motor and behavioural choice tasks (Adams et al., 2015; Cullen et al., 2018; Limanowski & Friston, 2020; Smith et al., 2020). While we used relatively simple learning models to characterise Bayesian inference through eye position, modelling approaches based on partially observable Markov decision processes (Smith et al., 2022) can also be used to model active inference and condition action choices on anticipated future consequences of actions (expected free energy). These models may be effective in explaining the behaviour of eye movements in more dynamic tasks where anticipated movement capabilities influence action selection.

Notably, results did not support our second hypothesis, which stated that gaze variability prior to stimulus onset would track predictive uncertainty (H2), as there was very little difference in variability between conditions. This result contrasts with that of Bakst & McGuire (2021) who reported greater variance in eye position under greater uncertainty (e.g. 50/50 compared to 90/10 conditions), positing that eye movements therefore encoded both the central tendency *and* precision of predictions. A possible reason for this difference is the fewer trials in our task, which would prevent particularly precise beliefs from developing. When examining the relationship between the parameters from the HGF2 models and gaze variability (on a per subject basis) we did, however, observe a relationship. Beta weights were significantly different from zero (*d* = 0.46), suggesting that gaze variability may, in fact, be indicative of belief precision as suggested by Bakst & McGuire (2021). The absence of this relationship in the between-condition comparisons may thus indicate that *perceived uncertainty* (as estimated by the models) was more important than *objective uncertainty* (as manipulated by the experimental conditions).

Our third hypothesis (H3) was that participants would use a MSE rather than MAP strategy, and therefore, predictive gaze position would be more extreme (further from centre) under more strongly biased conditions (e.g. 90% left location compared to 70% left). This result would support a Bayesian prediction error minimization explanation of oculomotor control (Adams et al., 2015; Parr et al., 2021). Results instead suggested that MAP, or perhaps some other more conscious strategy, was being employed. On the right-hand side of the task space, gaze positions for the most biased condition (10/90) were more extreme than the next most biased (30/70), consistent with a MSE strategy. This did not, however, hold for the left-hand side, where no significant difference was present for 90/10 versus 70/30, consistent with a MAP strategy. This disparity might reflect the observed asymmetry in task difficulty between left and right ball trajectories.

Plots of the distributions of predictive gaze position (see Fig. 4 top) showed that immediately prior to ball onset, participants were not fully adhering to either a MSE or MAP strategy. Participants did not adopt intermediate positions between the two locations that matched the mean of the generative distribution, consistent with a MSE strategy. Instead, they were predominantly directed to either of the projection locations (left or right) or in the centre. This suggests that even though participants were learning the probability distributions (as shown by the differences in predictive gaze location and mean gaze position), at the moment of release they were opting to either make a best guess or to ‘hedge their bets’ and look centrally, relying on peripheral vision for ball tracking (as has been reported in some sporting tasks (Klostermann et al., 2020; Vater et al., 2017, 2020)). This is partially consistent with a MAP approach, but the frequency of looking centrally also indicates that participants were not always making a ‘best guess’. The fitting of the learning models to eye position data did, however, indicate that Bayesian inference provided a better explanation of the updating of eye position over trials than associative learning, thereby providing further evidence that people were learning the task in an approximately Bayesian fashion. Overall, it appears that the *searching of the task space* prior to ball release followed a Bayesian or MSE strategy and Bayesian inference provided a good model for task learning, *but* at the moment of ball release participants mostly adopted a ‘best guess’ or a ‘look centrally’ strategy (Vater et al., 2020). Anecdotally, many participants reported consciously adopting this centre-looking peripheral-tracking strategy afterwards, and the functional utility of these behaviours (and their relationship with underlying visuomotor networks) therefore warrants further examination.

This deviation from Bayes-optimal gaze control might reflect the importance of the action component of the task (and the close intertwining of perception and action); if the primary aim was simply to observe the ball as clearly as possible, a MSE strategy may well have been used (as was demonstrated in Bakst & McGuire, 2021), but other factors such as the potential to use peripheral vision for tracking or the challenges of backhand interceptions may have led to the alternative gaze control strategies observed here. Subramanian et al. (2023) report that during a saccadic suppression displacement task in which participants had to judge whether a target had moved during a saccadic shift, participants displayed aspects of both Bayesian and ‘anti-Bayesian’ behaviour. Specifically, human participants were Bayesian for continuous reports of object displacement but anti-Bayesian for categorical reports (shift v no shift). Notably, it was found that a discriminative learning rule model (i.e. learning the boundary or decision surface separating different categories) better accounted for behaviour in these cases. Hence, while observers may display aspects of Bayes-optimal perception, it appears that alternative approaches may also be implemented during active perception tasks.

While the focus of this work was not on performance, the difficulty in determining the cost function associated with incorrect prediction and mis-alignment of the fovea with the release point is a clear limitation. As discussed above, task performance was heavily influenced by the response difficulty (forehand/backhand), so the relationship between interception and correct prediction was unclear. The performance analysis (see supplementary files: https://osf.io/9degj) indicated that release location (left/right) was a large and significant predictor of performance, while the distance between eye position and ball position at release was not. In Bayesian decision models (Körding & Wolpert, 2006), the relative values of different outcomes (i.e. cost functions) are used to scale action selection, so sensorimotor decisions are not just based on the most likely outcome but also the cost of different choices. Here, we could not determine the value of incorrectly predicting the wrong location but given that participants did adjust predictions in line with the condition probabilities, it suggests that there was some value in anticipating correctly, even if this was not detectable in our results.

**Fig. 8:**
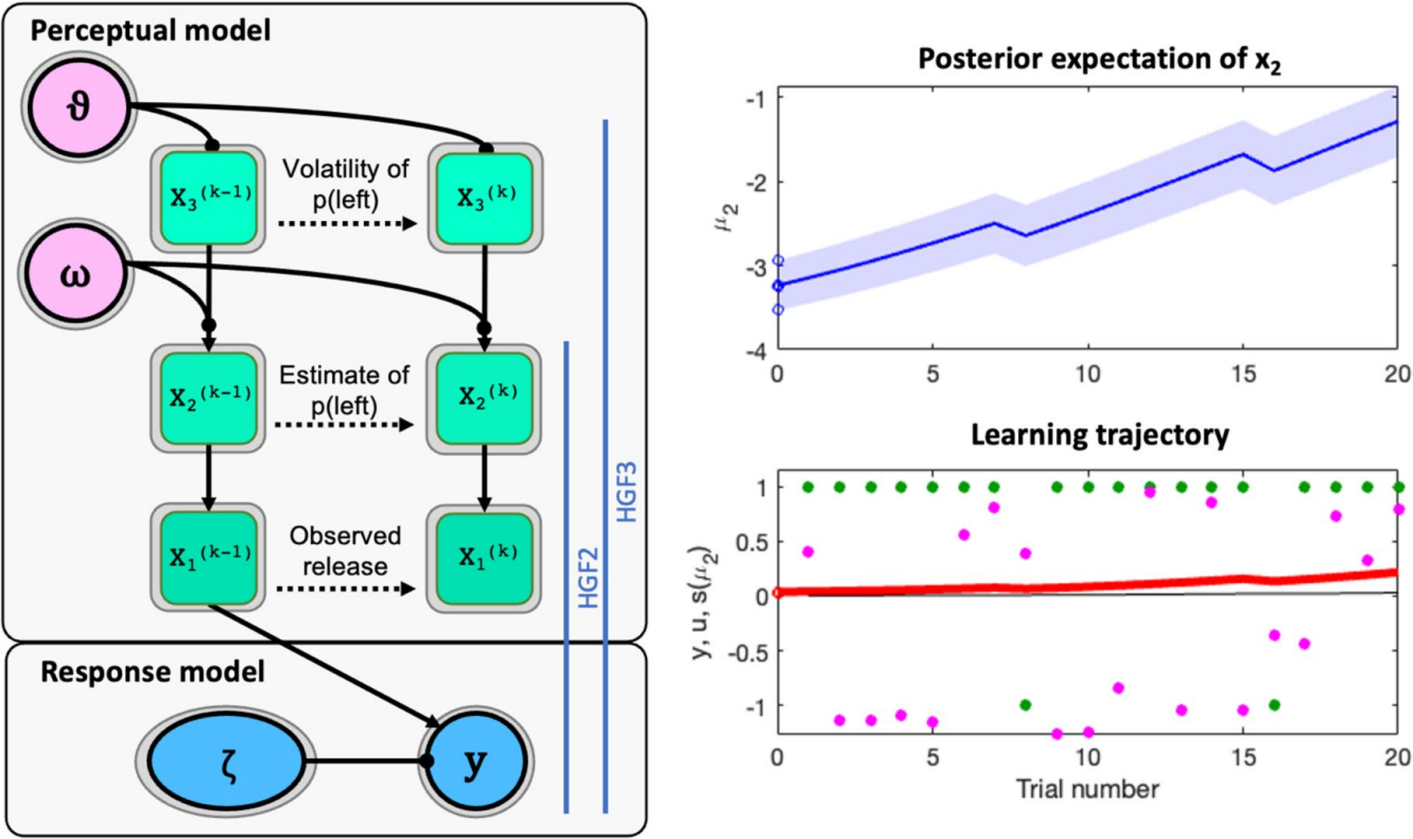
The HGF model. Note: Left: Schematic of the structure of the HGF. The perceptual model is described via beliefs (*x*) represented at multiple layers that evolve across time (*k*), scaled by variance parameters (ω, ϑ). The response model characterises the mapping between beliefs (*x*) and responses (*y*) using the ‘inverse decision temperature’ parameter (ζ), which controls the extent to which mapping from beliefs to responses is fully deterministic or more exploratory. Right: Example of the learning trajectory taken from a single participant. The lower panel shows eye position (fuchsia dots), observations (green), learning rate (fine black), and posterior expectation of input s(μ2) (red). The upper panel shows the evolving belief about x_2_ over trials

Our final hypothesis (H4) predicted that participants would exhibit greater physiological surprise responses (pupil dilations) to stimuli that were theoretically more improbable. As expected, the largest task-evoked pupil dilations were indeed observed for the 10% and 30% balls (see Fig. 8), which indicates that participants were most surprised by probabilistically salient sensory events. Dilations for the 90% and 70% balls were, however, larger than for the 50% balls—the opposing direction of effect to our hypotheses. The 90% balls should theoretically have been the least surprising events, yet they still evoked a larger response than balls in the 50% condition. One possible reason is that the number of trials used here was the minimum amount where participants could have learned the true distributions (Berniker et al., 2010), so the beliefs about what was ‘normal’ and ‘surprising’ would not have been as strongly learnt as tasks with greater numbers of trials (e.g. Bakst & McGuire, 2021). For instance, after a series of balls from the left-hand side in a 90/10 condition, participants might have been expecting a ball from the right, so could have actually been *more* surprised when balls continued to come from the left. The analysis of the relationship between pupil responses and parameters from the HGF2 model provided further support for a link with encoding of surprise and uncertainty, and a link with the locus coeruleus-norepinephrine system (Joshi et al., 2016). Consistent with this role, no relationship between pupil responses and the central tendency of beliefs about ball projection location (μ_2_) emerged in the dataset, but a relationship with *precision* of beliefs about release location (σ_2_) as well as the rate of change in beliefs (ω) was observed. These findings replicate previous results reported by Harris et al. (2022a) and show that trial-by-trial changes in neurophysiological activity align with internal expectations about environmental uncertainty and stability.

Our results provide an extension to previous work on active inference that has predominantly used simple behavioural choice tasks and are therefore more generalizable to dynamic visuomotor tasks than many previous studies. There are some important constraints on generality because this task was still highly simplified compared to real interceptive skills during tennis or squash. Our participant sample consisted of undergraduate students who were not task experts and extensive prior experience in either this game or related interceptive tasks could influence performance. There is, however, no reason to suppose that the general principles of Bayesian inference and their relationship with oculomotor control should be strongly influence by our participant sample.

## Conclusions

In this work, we examined how probabilistic generative models are used to regulate dynamic visuomotor responses, and whether human gaze behaviours exhibit the core principles of Bayesian inference that are proposed in recent neurocomputational theories (Adams et al., 2015; Friston et al., 2012; Parr & Friston, 2019; Parr et al., 2021). The findings suggested that observers did indeed encode the underlying probabilistic relationships of the task, but that control of the gaze system did not fully adhere to these rules. Instead, we observed possible trade-offs between accurate prediction-making and action capabilities, and the use of gaze strategies that did not necessarily follow ‘optimal’ Bayesian principles. Future work should examine whether the trade-offs between action capabilities and environmental probabilities can also be explained as a prediction error minimization process.

## Data Availability

All relevant data and code is available online from: https://osf.io/tgx6r/.

## Footnotes

1 We also pre-registered a by-condition comparison of interceptive performance, but successful interception was so heavily influenced by handedness (forehand easier than backhand) that the comparison of the probability conditions was confounded by the difficulty of executing the swing. This analysis is, therefore, not informative and only reported in the supplementary files (https://osf.io/gprbu/).

2 To explore whether this bias was only present in left-handed participants, we generated plots of gaze position split by handedness, which are available in the supplementary files (https://osf.io/7x6fu).
